# Hints on the Hypodermic Use of Morphia

**Published:** 1878-07

**Authors:** 


Hints on the Hypodermic use of Morphia.—A death occurred not long since in Washington City from the hypodermic injection of the third of a grain of sulphate of morphia, in the case of an adult laboring under pneumonia. A distressing cough had led to the administration of several doses of Dover's powder during the day, which failing to give relief, the morphia was introduced in the evening. Narcosis speedily ensued, and in spite of well-judged antidotal efforts, death followed in a few hours. The patient had frequently used opium and morphia with no bad effect.

This is not the only instance that has come to my knowledge of death attributed to the hypodermic injection of morphia. Some years ago a prominent individual in one of our California cities lost his life under similar circumstances; and the event threw so much discredit on the hypodermic use of morphia, that the practice has been almost abandoned in that locality. I have heard of similar occurrences in the Atlantic States, with the same effect of discouraging the plan of treatment. As a question in therapeutics, it is well to inquire how far such results should deter from the practice. That there are exceptions, and rare exceptions, to the general rule, will not be disputed. Doubtless the fatal result has been due in some cases to certain conditions of the brain, lungs or heart. The instance to which allusion has been made as occurring in this State, was ascribed to the cardiac disease under which the patient labored. I have known another instance of death succeeding the injection of about the third of a grain, the patient having some cerebral disorder, which seemed to be developed into apoplexy by the treatment. In the Washington case, the lungs were the vulnerable point through which the toxic action was brought to bear on the brain. But the probability is that in a small number of exceptional cases, the hypodermic injection of a full dose of morphia, say from one-third to one-half grain, will produce a fatal result, when no explanation can be given. The same thing is known to happen from opiates given by the stomach. So the inhalation of chloroform, and even of ether, will occasionally kill, without possible explanation. We talk of idiosyncrasy in such cases, and there we must stop. To throw away a valuable remedy or plan of treatment because of such rare fatalities, were irrational. There are many conditions of serious disease which can be relieved in no other way, as promptly if at all, as by injecting morphia beneath the skin. The practitioner who discards the process entirely, runs the risk of death to his patients fifty times that he may avoid a single risk.

As to the dose, it is not worth while to attempt to relieve severe pain with less than from one-third to one-half grain. Writers mention the eighth of a grain as the ordinary quantity; but my experience is that this small quantity is very inefficient and unsatisfactory. Those who limit themselves to so small an amount, will fail so frequently as to induce them to abandon the practice. I add a few hints which some of my readers may find useful, even though they may not be new:

1. Avoid it in congestion and inflammatory conditions of the brain.

2. Avoid it in pulmonary congestion, and where dyspnea is not the result of spasm.

3. Avoid it in acute inflammatory affections of the heart and pericardium.

4. Avoid it in high febrile excitement.

5. Avoid puncturing a vein. The effect of introducing morphia into a vein is instantaneous. It is possible that the evil results were due in some cases to the entrance of the narcotic into a small vein—so small and deep as to escape observation.

6. Avoid a deep puncture, unless there is a special purpose to be accomplished by depositing the narcotic deep in the tissues.

7. Introduce the liquid slowly and not by sudden projection, as is sometimes done. Spend four of five seconds in driving the piston home.

8. Require the patient to lie down and remain quiet after the operation. There is some risk in moving about, independently of the nausea which follows. The syringe should be used in the patient's chamber, and not in the doctor's office.

I may add, in regard to the appliance in cardiac cases, that, according to my experience, there is no condition of human suffering to which it is more applicable than in the orthopnea of cardiac asthma. In fact, it is the remedy, *par excellence*, for the paroxysm of spasmodic asthma, from whatever cause.

By observing the foregoing cautions, I think the risk of injury from the hypodermic use of morphia will be reduced to a minimum, whilst the physician will be armed with an instrumentality for the relief of pain, the value of which is beyond calculation.

A singular phenomenon, which I am not able to explain satisfactorily, has come under my observation in some exceptional cases, namely, the aggravation of pain immediately after the injection. The patient complains that the pain is suddenly augmented, say in twenty or thirty seconds after the injection; but the aggravation is brief, passing away in less than a minute, and is succeeded by the proper action of the drug.

Another effect is much more common—a distressing sensation which takes the place of the pain, and causes the patient to complain that he, or more frequently she, feels as if dying. "I feel as if I was dying," is the expression. It is the stepping-stone, so to speak, between pain and hypnotism. It lasts only for a fraction of a minute, and vanishes with the accession of the hypnotic stage. I confess to a feeling of anxiety whenever it occurs, and am careful to require the patient to keep quiet in the horizontal posture.—*Pacific Medical and Surgical Journal.*

